# A retrospective feasibility study of biweekly, reduced-dose docetaxel in Asian patients with castrate-resistant, metastatic prostate cancer

**DOI:** 10.1186/s12894-017-0253-z

**Published:** 2017-08-22

**Authors:** Hae Su Kim, Ji Yun Lee, Su Jin Lee, Ho Yeong Lim, Hyun Hwan Sung, Hwang Gyun Jeon, Byong Chang Jeong, Seong Il Seo, Seong Soo Jeon, Hyun Moo Lee, Han-Yong Choi, Se Hoon Park

**Affiliations:** 1 Division of Hematology-Oncology, Department of Medicine, Samsung Medical Center, Sungkyunkwan University School of Medicine, 81 Irwon-Ro Gangnam-gu, Seoul, 135-710 South Korea; 2 Department of Hematology-Oncology, Department of Medicine, Veterans Health Service Medical Center, Seoul, South Korea; 3 Department of Urology, Samsung Medical Center, Sungkyunkwan University School of Medicine, Seoul, South Korea

**Keywords:** Castrate-resistant prostate cancer, Docetaxel, Biweekly

## Abstract

**Background:**

The aim of this retrospective study was to evaluate the clinical outcomes of reduced dose, biweekly docetaxel chemotherapy for Korean patients with castrate-resistant prostate cancer (CRPC).

**Methods:**

We retrospectively reviewed the medical records of 48 patients with metastatic CRPC who were treated with a biweekly regimen (intravenous docetaxel 40 mg/m^2^ on day 1 plus prednisolone 5 mg twice daily) between 2012 and 2015 at Samsung Medical Center (Seoul, Korea). Prior to the adoption of a biweekly regimen in Oct 2013, our institutional standard chemotherapy was docetaxel 75 mg/m^2^ every 3 weeks for patients with CRPC (*n* = 24). After Oct 2013, all chemotherapy-naïve patients with CRPC received a 40 mg/m^2^ biweekly regimen (*n* = 24). The primary end point was a PSA response, defined as a greater than 50% decline in PSA level from baseline.

**Results:**

The baseline characteristics of the patients in the two treatment groups were similar. The most common cause of treatment discontinuation was disease progression, which was exhibited by 17 patients (71%) in the 3-weekly group and 20 (75%) in the biweekly group. PSA responses were observed in 12 (50%) and 11 (46%) patients in the 3-weekly and biweekly groups, respectively *(p* = 0.683). Time to treatment failure (TTTF, 4.5 vs 3.9 months) and time-to-progression (TTP, 5.0 vs 4.2 months) were not significantly different between the 3-weekly and biweekly groups.

**Conclusions:**

Within the limitations of a retrospective study, the biweekly reduced dose docetaxel regimen was active and well-tolerated in Korean patients with metastatic CRPC.

## Background

Prostate cancer is one of the most rapidly rising malignancies in Korea [1]. In patients with advanced or metastatic disease, androgen-deprivation therapy (ADT) or surgical castration is regarded as the standard treatment. After years of treatment, however, medically or surgically castrated prostate cancer eventually transforms into castration-resistant prostate cancer (CRPC) [2]. Although the prognosis of patients with CRPC has typically been very poor [3], two randomized clinical trials demonstrated a survival benefit with docetaxel chemotherapy in patients with metastatic CRPC [4, 5]. Docetaxel is usually administered at a dose of 75 mg/m^2^ every 3 weeks based on the results from the TAX-327 study [5, 6], in which 3-weekly docetaxel conferred a clear survival benefit over mitoxantrone (median, 19.2 vs 17.8 months; *P* = 0.004), but was also associated with significant hematologic toxicity.

As a result of these two landmark trials, docetaxel was approved in Korea for the treatment of CPRC. Moreover, a retrospective study [7] demonstrated that the standard regimen (docetaxel 75 mg/m^2^ every 3 weeks plus prednisolone 5 mg twice daily) was feasible in Asian patients with CRPC and yielded a manageable toxicity profile. However, based on a pharmacokinetics study conducted in Japan [8] and the belief that treatment safety and tolerability are indispensable in the treatment of solid tumors in a palliative setting [7], docetaxel is most commonly administered in Asian countries at a lower dose (60 mg/m^2^ every 3 weeks).

Another way to circumvent docetaxel-induced hematologic toxicity is to use different administration schedules, such as weekly or biweekly regimens. In support of this strategy, weekly administration of docetaxel has been shown to yield lower hematologic toxicity than the standard 3-weekly regimen [9]. Moreover, grade 3 or 4 neutropenia occurred in 75% of all Asian patients with CRPC who were treated with 3-weekly docetaxel [10]. Furthermore, the median survival duration in the weekly docetaxel arm of the TAX-327 study [6] was shorter than in the mitoxantrone arm (median, 17.8 vs 16.3 months; *p* = 0.09), although this difference was not significant. In contrast, biweekly administration of docetaxel 50 mg/m^2^ resulted in a longer time-to-treatment failure (TTTF; median, 5.6 vs 4.9 months; *p* = 0.014) than 3-weekly docetaxel [11]. As expected, the biweekly regimen was better tolerated than the 3-weekly docetaxel regimen; importantly, efficacy was not compromised.

Based on these considerations, in Oct 2013 we adopted a biweekly low dose docetaxel regimen (40 mg/m^2^ every 2 weeks) as an institutional standard chemotherapy regimen for patients with chemotherapy-naïve CPRC. Here we retrospectively investigated and compared the clinical outcomes of biweekly 40 mg/m^2^ docetaxel plus prednisolone with those of 75 mg/m^2^ docetaxel every 3 weeks in korean patients with CRPC.

## Methods

### Patients

We retrospectively collected and reviewed the medical records of 48 patients with metastatic CRPC who were consecutively treated with docetaxel plus prednisolone as the first-line chemotherapy regimen between March 2012 and February 2015. Patients with histologically-proven adenocarcinoma of the prostate whose disease had progressed after maximal ADT were eligible for the study. Disease progression was defined as 1) radiologic evidence of a new metastatic lesion or aggravated measurable disease, or 2) serial increases in the prostate-specific antigen (PSA) level on 2 or more occasions at least 2 weeks apart. Patients were required to have a castrate level of serum testosterone while receiving ADT, an Eastern Cooperative Oncology Group (ECOG) performance status of 0 to 2, and adequate major organ functions. We excluded all patients who were enrolled in clinical trials to ensure that the study population reflected our daily clinical practice. The choice of biweekly docetaxel was solely at the discretion of the treating oncologists. Other exclusion criteria were as follows: (1) prior chemotherapy for advanced or metastatic disease, (2) histologic evidence of neuroendocrine carcinoma, (3) another malignancy within 5 years, and (4) inappropriate laboratory findings or any severe comorbidity.

### Procedures

Prior to the adoption of a biweekly regimen in Oct 2013, our institutional standard chemotherapy was docetaxel 75 mg/m^2^ every 3 weeks for patients with CRPC. After Oct 2013, all chemotherapy-naïve patients with CRPC at our institution received a biweekly regimen. Docetaxel was administered intravenously over 1 h on day 1 with dexamethasone and anti-emetics. Oral prednisolone 5 mg was administered twice daily from day 1 and continued throughout treatment. Supportive care, including the administration of blood products, bisphosphonates, and the use of analgesics was given if judged appropriate by the treating physicians. Before initiating the first dose of docetaxel, a complete history was taken from each patient. In addition, complete blood counts, serum chemistry analyses, chest x-rays, bone scans, and computed tomography (CT) scans of all involved sites were performed. Patients were seen every 2 or 3 weeks; during these visits, a brief history was taken, a physical examination was performed, and adverse events, blood counts, and PSA levels were assessed. In both groups, treatment was repeated on an outpatient basis and continued until objective disease progression, unacceptable toxicity, deterioration of clinical conditions, or patient refusal. Radiologic responses were evaluated every 6 weeks (3-weekly group) or 8 weeks (biweekly group) by bone scan, chest and abdominopelvic CT, or the same tests that were used for initial tumor staging. Adverse events were collected and graded according to the National Cancer Institute criteria (CTCAE) version 4.0.

### Statistical analysis

The primary end point was a PSA response, defined as a ≥ 50% decline in the PSA level from baseline with no clinical or radiologic evidence of disease progression. PSA progression was defined as an increase of ≥25% and ≥2 ng/ml above the nadir that was confirmed by a second value 3 or more weeks later. If no decline from baseline was observed, PSA progression was defined as an increase of ≥25% and ≥2 ng/ml after 12 weeks according to the Prostate Cancer Working Group 2 (PCWG2) criteria [12]. Secondary end points included TTTF, time to progression (TTP), duration of PSA response, and toxicity profile. TTTF was defined as the time from the first administration date to the date of disease progression (PSA or radiologic progression), unacceptable toxic effects, death, or discontinuation of chemotherapy for any reason. TTP was defined as the time from the first administration date to the date of disease progression or death. TTTF and TTP were calculated using the Kaplan-Meier method. Categorical variables were compared using Pearson’s chi-square test or Fisher’s exact test. Continuous variables were compared with the Mann-Whitney U test. TTTF and TTP were calculated using the Kaplan-Meier method and compared using the log-rank test. An unstratified Cox regression model was used to estimate hazard ratios (HRs) with 95% confidence intervals (CIs). All *p* values were two-sided, with *p* < 0.05 taken to indicate statistical significance. All analyses were performed using R for Windows, v2.11.1 (R Core Team, Vienna, Austria; http://www.Rproject.org).

## Results

### Patient characteristics

From March 2012 to Feb 2015, 58 patients with CRPC underwent screening, 48 (83%) of whom were eligible for the current study. Each group contained 24 patients. The patient baseline characteristics were not significantly different between the two treatment groups (Table 1). The median patient age was 68 years (range, 52–84 years) and the most common site of metastasis was bone (100%), followed by lymph node (58%) and bladder (8%). A total of 42 (88%) patients had received prior hormonal therapy, and 8 (17%) patients had undergone prostatectomy. The median follow-up duration after the first chemotherapy treatment was 11 months (range, 2.5–36.3 months).

**Table 1:** Baseline characteristics according to docetaxel regimens

Characteristic	3-weekly docetaxel (*n* = 24)	2-weekly docetaxel (*n* = 24)	*P* value
Median age (years) (range)	69.5 (52.3–83.9)	64.6 (56.6–80.0)	0.110
Site of metastasis			
Bone	24 (100%)	24 (100%)	1.000
Liver	2 (8.3%)	1 (4.2%)	0.500
Lung	1 (4.2%)	2 (8.3%)	0.500
Lymph node	10 (41.7%)	18 (75%)	0.019
Bladder	1 (4.2%)	3 (12.5%)	0.609
Gleason score			0.190
≤ 7	5 (20.8%)	3 (12.5%)	
8	6 (25.0%)	5 (20.8%)	
≥ 9	7 (29.2%)	16 (66.7%)	
Unknown	6 (25.0%)	0 (0%)	
Median PSA (ng/mL)			
Baseline	34.7	31.2	0.279
Nadir	15.1	11.6	0.279
Previous therapy			
Prostatectomy	4 (16.7%)	4 (16.7%)	0.500
Prostate radiotherapy	2 (8.3%)	1 (4.2%)	0.500
Total dose (mg/m^2^), median	395	320	0.011
Total # cycles, median (range)	6 (1–11)	8 (2–23)	0.023
Mean dose (mg/m^2^) at each cycle, median	68 (58–75)	40 (35–40)	<0.001
Initial dose (mg/m^2^), median	75 (60–75)	40 (35–40)	<0.001

*Abbreviations*: *PSA* prostate-specific antigen

### PSA response

A PSA response was observed in 12 (50%) patients in the 3-weekly group and 11 (45.8%) patients in the biweekly group (*p* = 0.683) (Table 2). Among the patients with a PSA response, the median time to PSA response was 1.4 months in both groups (*p* = 0.839). The median response durations were 4.0 and 3.7 months in the 3-weekly and biweekly groups, respectively (*p* = 0.342).

**Table 2:** Summary of primary and secondary outcomes

Outcome	3-weekly docetaxel (95% CI)	2-weekly docetaxel (95% CI)	*P*-value	Hazard ratio (95% CI)	*P*-value
Overall PSA response			0.683		
Objective response^a^	12 (50.0%)	11 (45.8%)	..	..	..
Stable disease	10 (41.7%)	9 (37.5%)	..	..	..
Disease progression	2 (8.3%)	4 (16.7%)	..	..	..
Median response duration months, (95% CI)	4.0 (3.2–8.1)	3.7 (2.7–6.0)	0.342		
Median TTTF, months, (95% CI)	4.5 (3.1–5.9)	3.9 (3.2–4.6)	0.542	1.2 (0.7–2.2)	0.551
Median TTP, months, (95% CI)	5.0 (3.9–6.1)	4.2 (3.5–4.9)	0.530	1.2 (0.6–2.3)	0.536

*Abbreviation*: *CI* confidence interval; *PSA* prostate-specific antigen; *TTTF* time to treatment failure; *TTP* time to progression; *PSA* prostate-specific antigen

Medians and 95% CIs were estimated from Kaplan-Meier analyses

^a^Objective responses include only partial responses; no complete responses were observed

### TTTF and TTP

**Fig. 1:**
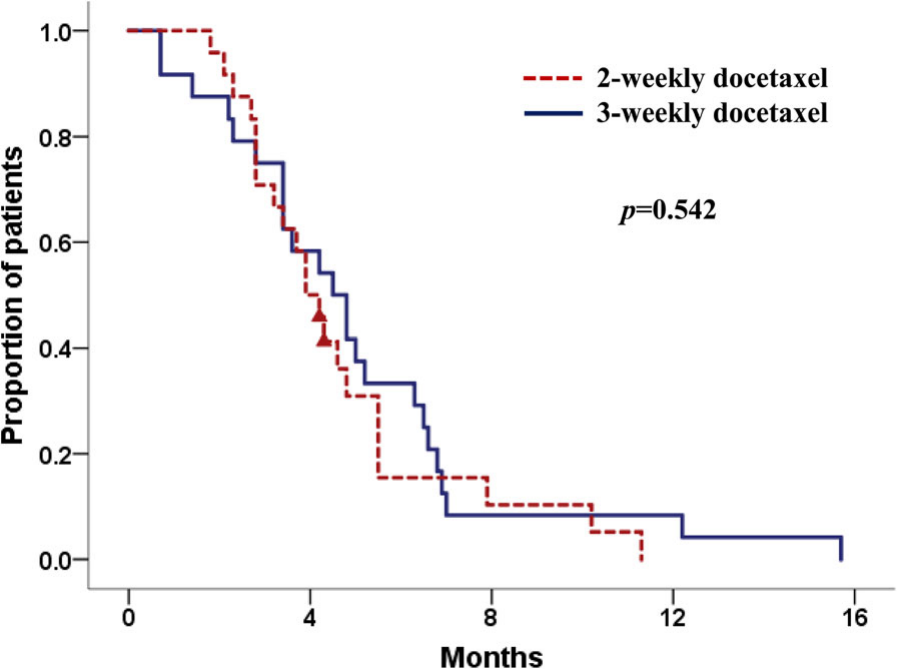
Kaplan-Meier survival analysis according to time to treatment failure (log-rank *p* = 0.542)

**Fig. 2:**
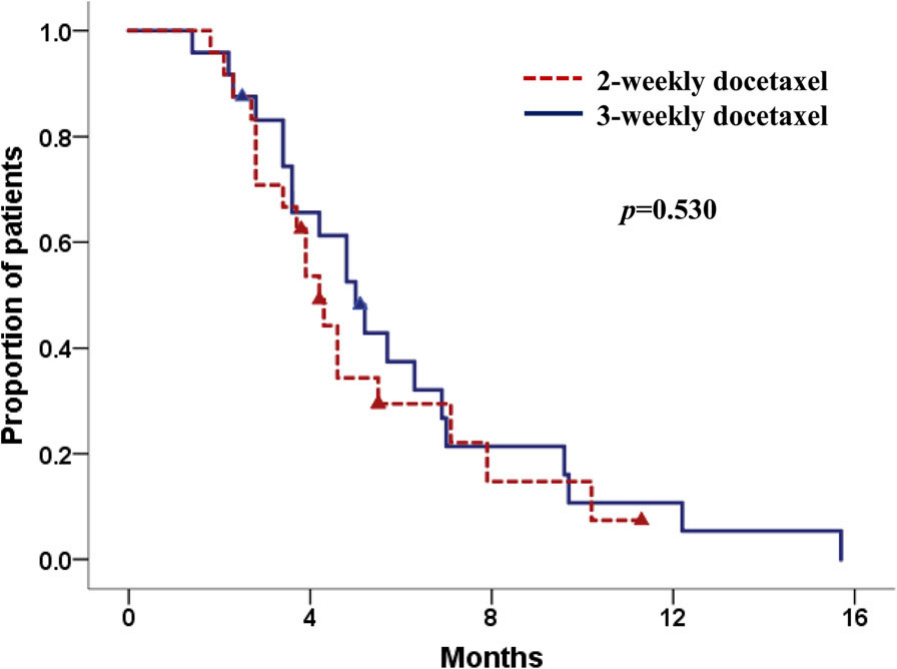
Kaplan-Meier survival analysis according to time to progression (log-rank *p* = 0.530)

The median TTTF was longer in the 3-weekly group than in the 2-weekly group; however, this difference was not significant (4.5 months, 95% CI 3.1–5.9 in the 3-weekly group vs 3.9 months, 95% CI 3.2–4.6 in the 2-weekly group, *p* = 0.542; Fig. 1). Similarly, the TTP were not significantly different between the two groups (5.0 months, 95% CI 3.9–6.1in the 3-weekly group vs 4.2 months, 95% 3.5–4.9 in the 2-weekly group, *p* = 0.530; Fig. 2). At the time of data cut-off (July 2015), six (25%) patients in the 3-weekly group and four (17%) patients in the 2-weekly group had died. The median OS had not been reached in either group.

### Toxicities

The median numbers of cycles per patient were six (range, 1–11) in the 3-weekly group and 8 (range, 2–23) in the 2-weekly group (Table 1). The most common cause of treatment discontinuation was disease progression (17 [71%] in the 3-weekly group; 18 (75%) in the 2-weekly group), followed by adverse events (4 [17%] and 1 [4%]), patient refusal of treatment (2 [8%] and 0), and unknown reasons (1 [4%] and 3 [13%]). The median cumulative doses of docetaxel were 395 mg/m^2^ in the 3-weekly group and 320 mg/m^2^ in the 2-weekly group. Fourteen (58%) patients in the 3-weekly group and 4 (17%) in the 2-weekly group required dose reduction. Toxic effects requiring dose reduction included fatigue (61%), neutropenia (22%), thrombocytopenia (11%), and nausea (6%). The hematological and non-hematological toxic effects for each group are listed in Table 3. No patient died from any therapy-related toxic effect. The most common hematologic toxicity was anemia. Four patients (17%) who received 3-weekly docetaxel had grade 3–4 neutropenia; among them, 3 (13%) experienced neutropenic infections. In contrast, no patient who received 2-weekly docetaxel exhibited febrile neutropenia. Eight (33%) patients in the 3-weekly group and 3 (13%) in the 2-weekly group had grade 3–4 fatigue.

**Table 3:** Treatment-related adverse events

	3-weekly docetaxel (*n* = 24)		2-weekly docetaxel (*n* = 24)	
	Grade 1–2	Grade 3–4	Grade 1–2	Grade 3–4
Neutropenia	7 (30%)	4 (17%)	5 (21%)	0
Febrile neutropenia	..	3 (13%)	..	0
Anemia	23 (96%)	1 (4%)	15 (63%)	1 (4%)
Thrombocytopenia	4 (17%)	2 (8%)	2 (8%)	0
Fatigue	8 (33%)	8 (33%)	5 (21%)	3 (13%)
Peripheral neuropathy	0	0	1 (4%)	0
Allergic reaction	0	0	1 (4%)	0
Arthralgia	8 (33%)	0	6 (25%)	0
Diarrhea	6 (25%)	0	7 (29%)	0
Nausea	4 (17%)	1 (4%)	5 (21%)	0
AST elevation	1 (4%)	0	0	0
ALT elevation	1 (4%)	0	0	0

## Discussion

The purpose of the present retrospective study was to evaluate the feasibility of biweekly docetaxel 40 mg/m^2^ in patients with metastatic CRPC. Patients who received docetaxel every 2 weeks showed similar PSA responses, TTTFs, TTPs, and fewer occurrences of grade 3–4 adverse events compared with patients who received docetaxel 75 mg/m^2^ every 3 weeks.

Several treatment options are available for men with progressing metastatic CRPC, including docetaxel plus estramustine [4], cabazitaxel plus prednisone [13], abiraterone plus prednisone [14], enzalutamide [15], radium-223 [16], and sipuleucel-T [17]. However, docetaxel with prednisone has been the standard first-line drug for men with metastatic CRPC for more than 10 years, based on two landmark studies showing a 20–30% improvement in OS compared with mitoxantrone chemotherapy [4, 5]. The GETUG-AFU-15 and CHAARTED studies, both of which assessed the efficacy and tolerability of ADT (with or without docetaxel), also suggested that ADT plus docetaxel should be considered as a standard treatment in men with metastatic hormone-sensitive prostate cancer [18, 19].

Now that docetaxel has been approved as a first-line treatment for metastatic CRPC, it is particularly relevant to determine whether administering a lower dose of docetaxel over a shorter interval yields improved efficacy and tolerability compared with the standard regimen of 75 mg/m^2^ docetaxel every 3 weeks. The TAX 327 trial showed significantly longer survival for patients treated with 75 mg/m^2^ docetaxel every 3 weeks compared with 12 mg/m^2^ mitoxantrone every 3 weeks (OS 19.2 months vs 17.8 months, respectively); however, patients treated with 30 mg/m^2^ docetaxel once weekly (OS 17.8 months) did not exhibit any significant difference in survival compared with patients treated with the mitoxantrone regimen [6]. Although the 3-weekly docetaxel regimen yielded a slightly longer OS than the weekly docetaxel regimen, 32% of the 332 patients who received 3-weekly docetaxel experienced grade 3–4 neutropenia, whereas only 2% of the 330 patients who received weekly docetaxel had grade 3–4 neutropenia [5].

Few studies have determined the optimal schedule for docetaxel-based chemotherapy so that it is both efficacious and safe. In one phase III study, 170 patients were treated with docetaxel 50 mg/m^2^ administrated every 2 weeks, whereas another 176 patients were treated with docetaxel 75 mg/m^2^ every 3 weeks [11]. Although the total doses in both groups were identical over the 12 weeks, 2-weekly administration was found to be associated with significantly longer TTTF than 3-weekly administration (5.6 vs 4.9 months; HR 1.3, *p* = 0.014). Moreover, 2-weekly administration of docetaxel was also associated with improved OS and fewer occurrences of neutropenia, leucopenia, and febrile neutropenia. In another study, 94 patients with metastatic CRPC were treated with docetaxel 45 mg/m^2^ every 2 weeks with estramustine [20]. A PSA response was observed in 45 (53%) of the 84 patients, and the median time to PSA progression was 5.0 months. In another study by Karavasillis et al., 16 patients with metastatic CRPC were treated with a regimen of biweekly docetaxel 30 mg/m^2^, and 6 patients (38%) showed a PSA response over a median duration of 4.5 months [21]. The patient characteristics, TTTF, and PSA responses in both groups in the present study were highly similar to those in a previous phase III study [11]. In the previous study, 2-weekly 50 mg/m^2^ docetaxel resulted in a significantly longer TTTF than 3-weekly docetaxel; however, no significant differences were observed between patients treated with 2-weekly 40 mg/m^2^ docetaxel versus 3-weekly 75 mg/m^2^ docetaxel in present study. We observed similar PSA responses and TTTFs in the 2-weekly and 3-weekly docetaxel groups; this result may be due to the relatively small dose of docetaxel in the 2-weekly group and the small number of patients. Therapy-related adverse effects and comorbidities are common in elderly patients and play important roles in determining the optimal approach to palliative chemotherapy for individuals with CRPC [22]. Our results show that a 2-weekly docetaxel chemotherapy regimen is well tolerated, safe, and yields results comparable to those achieved with a 3-weekly docetaxel regimen.

The current study has several limitations. First, 17% of the patients treated with docetaxel were not included in the present study, leading to potential selection bias. Second, given the retrospective nature of the study, some of the data may be missing, especially data regarding adverse events. Finally, patient-reported outcomes, such as quality-of-life factors, could not be determined.

## Conclusions

In conclusion, a biweekly reduced dose docetaxel regimen is active and well-tolerated in Korean patients with metastatic CRPC. Thus, a biweekly docetaxel regimen should be considered as an additional option for Asian patients and men with comorbidities. Our results warrant further large prospective studies of biweekly reduced dose docetaxel.

## Abbreviations

ADT: androgen-deprivation therapy
CRPC: castration-resistant prostate cancer
PSA: prostate-specific antigen
TTP: time to progression
TTTF: time-to-treatment failure
